# The Use of Age-Friendly Technology in the Care of Older Adults: Two Implementation Case Studies

**DOI:** 10.1177/07334648251343513

**Published:** 2025-06-24

**Authors:** Soe Han Tha, Katrina Hough, Nhat Bui, Sarah Dulaney, Carla Perissinotto

**Affiliations:** 1 Division of Geriatrics, Department of Medicine, 8785 University of California, San Francisco, CA, USA; 2 Memory and Aging Center, Department of Neurology, University of California, San Francisco, CA, USA

**Keywords:** dementia, technology, geriatrics, access to care, age-friendly, aging

## Abstract

Telemedicine has become standard in health care, and telehealth access is increasingly important. Some populations such as older adults may not have the tools nor the understanding to successfully use telehealth. We describe two cases where GrandPad, a tablet designed for older adults, was implemented at the University of California, San Francisco in (1) a home-based primary care practice and (2) a dementia specialty clinic. Using the Consolidated Framework for Implementation Research, we assess the barriers and facilitators to implementing GrandPad and present user outcomes. Most participants found GrandPad easy and comfortable to use, though there were mixed results about connecting with the provider’s office. Our implementation science analysis demonstrates that GrandPad may best be used when there is otherwise no access to telehealth, where there are frequent visits, and a need for continuity of care. Successful implementation includes clear integration into existing clinic processes, understanding users’ abilities, reducing provider barriers, and fostering potential users’ interest.

**What this paper adds**

• This paper provides context-specific insights that can inform strategic telehealth implementation efforts in populations traditionally left out of telehealth use: older homebound adults and adults living with dementia.
• This paper describes key implementation principles for adding telehealth capabilities in primary care and specialty care clinics.

**Applications of study findings**

• Study findings can contribute to narrowing care gaps among older adults by identifying contexts in which the GrandPad tablet (or other devices specific to older adults) is most effective as a telemedicine tool.
• Study findings can guide implementation of telemedicine strategies for older adults, particularly those who are homebound and/or living with cognitive disorders.

## Introduction

### Current State of Telemedicine for Older Adults

Research on the implementation of age-friendly telemedicine for primary care and specialty care for older adults, particularly those with dementia, is increasing but still limited (Loizos et al., 2023; Nkodo et al., 2022; Qin et al., 2024; Yi et al., 2021). While there are studies on telemedicine’s use in occupational therapy and exercise for dementia patients, specific research on age-friendly technology for this group remains scarce (Park et al., 2022; Rhodus et al., 2023). This study is the first of its kind to evaluate the feasibility of using a tablet *specifically* designed for older adults as a telemedicine tool in clinics primarily serving older adults and those with cognitive disorders.

Currently, 2 million adults in the United States are completely homebound (i.e., have not left the home in the past month) or mostly homebound (i.e., have rarely left the home in the past month), with an additional 4.6 million being semi-homebound (i.e., only left the home with assistance or had difficulty or needed help leaving the home) (Ankuda et al., 2021; Ornstein et al., 2015; Ritchie et al., 2018). Only 12% of those completely homebound receive primary care at home. In parallel, at least 5.8 million adults are living with Alzheimer’s dementia, with millions having other dementia subtypes (2023 Alzheimer’s disease facts and figures, 2023; Matthews et al., 2019). With current demographic shifts, these numbers will increase. Behavioral symptoms, common in all types of dementia, often result in significant caregiver stress and illness, institutionalization, and reduced quality of life for the patient and caregiver (Hoe et al., 2009; Selwood et al., 2005). These distressing symptoms can be associated with higher caregiver burden, earlier long-term care placement, and higher hospital use (Brodaty & Donkin, 2009; Oesterhus et al., 2020; Schulz & Martire, 2004). These behaviors often result in patients becoming home-limited or homebound because of the challenge of bringing patients into in-person clinics. Therefore, there is a critical need to assess and treat dementia patients experiencing behavioral symptoms without the disruption of clinic transportation.

In the management and care of individuals with dementia, caregivers play a critical role given the high level of dependence that individuals with cognitive impairment may have on their caregivers. Dyadic recruitment, involving both patients and their caregivers, ensures that telehealth solutions are tailored to meet their interconnected needs, providing comprehensive support and enhancing care effectiveness. Additionally, care needs often arise between scheduled appointment times, and the amount of asynchronous care needs can be extraordinary.

Telemedicine can help to lower care burdens, to reduce the need for in-person visits, and to limit emergency department (ED) visits by enabling healthcare needs to be readily assessed virtually. Encouragingly, patient and caregiver telemedicine assessments for dementia care have been found to be consistent with in-clinic assessments (Yi et al., 2021). Healthcare providers can also quickly gain valuable insights and knowledge from these remote consultations, improving patient care quality. In a patient-and-caregiver-dyads study involving 60 older adults with dementia (conducted from March to mid-May of 2020 during the beginning of the COVID-19 pandemic), investigators evaluated medical visits by video telehealth visits as opposed to telephone only; data showed that with video telehealth visits, patients’ Montreal Cognitive Assessment (MoCA) and Quality of Life-Alzheimer Disease (QoL-AD) scores significantly improved, and caregiver burden decreased (Lai et al., 2020). For these reasons, supplementing and/or replacing in-person visits with telehealth has become increasingly necessary and has, in many cases, already become a standard practice due to the safety concerns brought forth by the COVID-19 pandemic. Despite this general trend, older adults, particularly those with dementia, represent a population that would significantly benefit from this transition due to their unique challenges and the pressing need for more accessible and effective telehealth solutions.

Despite some encouraging data, the COVID-19 pandemic and the rapid implementation of telehealth left many older adults and low-income adults without access given what has been described as a digital divide (Vogels, 2021; Wardlow et al., 2023). Additionally, many telehealth platforms are not designed for people with functional limitations and not adaptable for those with visual, hearing, or cognitive impairment (Wardlow et al., 2023; Yi et al., 2021). Although guidelines for telehealth and aging are being developed (such as the set of recommendations created by the Collaborative for Telehealth & Aging), significant challenges remain (The Center of Excellence for Telehealth and Aging; Loizos et al., 2023; Wardlow et al., 2023). For example, many telehealth platforms can be inaccessible due to a lack of Wi-Fi or mobile data plans. When access is not the issue, the executive function required to log into a meeting, turn on a camera, and locate the sound presents insurmountable barriers for others. Despite these barriers, satisfaction with telemedicine among older adults with cognitive impairment is growing. For instance, a study of 32 home-dwelling older adults with frontotemporal lobar dementia found that 90% of participants were both satisfied with telehealth and willing to continue telehealth utilization (Capozzo et al., 2020).

As an alternative to tablets that are readily available purportedly for all age-groups, GrandPads are data-enabled and Wi-Fi-capable tablets designed for older adults. Our study aimed to evaluate the feasibility of incorporating the GrandPad into two clinical settings at the University of California, San Francisco (UCSF): Care at Home (CAH), a home-based primary care practice for older adults, and the Memory and Aging Center (MAC), a specialty dementia care clinic. These settings were chosen because they serve populations facing significant barriers to telehealth, including homebound and/or cognitively impaired older adults who often lack digital literacy and access to conventional telehealth solutions. Evaluating the GrandPad in these settings addresses the gap in age-friendly telehealth implementation literature and provides insights for improving telehealth accessibility and effectiveness for these vulnerable populations.

**Figure 1.**
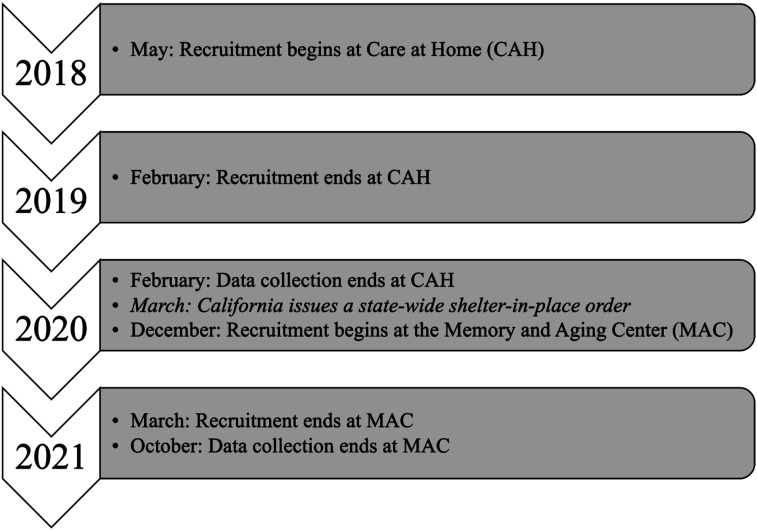
Project timeline.

Our study began at CAH prior to the COVID-19 pandemic, where the goal of the pilot was to target harder-to-reach CAH patients and those with high healthcare utilization. At MAC, our study began and continued throughout the first year of the pandemic (Figure 1) where the goal was to reach patients with dementia and neurobehavioral symptoms. We sought to determine barriers and facilitators to the implementation of the GrandPad in diverse clinical settings which use different telemedicine platforms.

## Methods

### Device Description

The GrandPad is designed for older adults and those with cognitive impairment or decreased visual or manual dexterity. It incorporates details that address common age-related challenges (e.g., vision and hearing and decreased touch sensitivity at the fingertips) with large, easy-to-press buttons and a simplified user interface displaying large icons and high-contrast text, making it more accessible for users with vision impairments and other functional impairments such as arthritis. The GrandPad also includes speakers that accommodate a higher volume level than the volume level of comparable devices. To help with accessibility, a device administrator (e.g., a family member or a caregiver) can remotely supervise and program the tablet, customizing content and making it engaging for users with cognitive impairment.

The GrandPad is commercially available and is used in healthcare settings to enable telehealth. Uniquely, as part of the monthly fee (approximately $60 during the study), a “concierge team” assists new users with the set-up of their device and troubleshooting, available 24/7. The 8-inch, 12.5-ounce tablet allows users to make video and audio calls (both via Zoom and the GrandPad’s native application), play music, view photos, and more with or without Wi-Fi due to the built-in data connectivity. During our study (2018–2021), GrandPad did not support the downloading of electronic health record platforms (e.g., EPIC and MyChart) or facilitate these platforms’ video calls. The tablet is “locked” purposefully so users do not receive unsolicited calls, thereby acting as a barrier to elder abuse, particularly scams. Each GrandPad user must “allow” the healthcare provider to be able to make calls directly to the user. The GrandPad concierge team can also assist with onboarding of new providers and users.

In our study, the GrandPads were provided free of charge to participants, funded by the UCSF Office of Population Health for CAH and the UCSF Osher Center for Integrative Medicine for MAC. This financial support was essential to eliminate cost barriers and facilitate the implementation of the GrandPad. At the time of the study, partners of GrandPad that were incorporating the tablet into care delivery included Programs of All-Inclusive Care for the Elderly (PACE) by Medicare and Medicaid (GrandPad expands footprint in health care, telehealth through partnerships with leading healthcare organizations, 2020). Since then, there has been growing interest in expanding telehealth coverage, with some Medicare Advantage plans and Medicaid programs beginning to explore coverage for telehealth devices and services (Telehealth Policy Updates, 2025).

### Description of Clinical Settings

**Figure 2.**
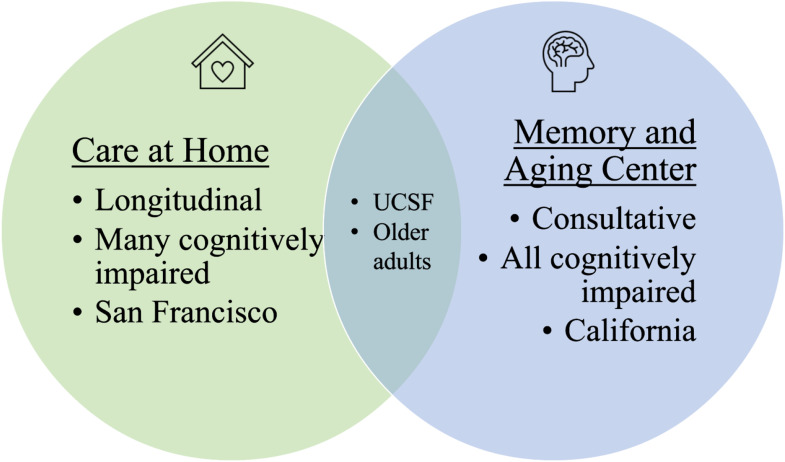
Site characteristics.

While both clinical settings serve older adults and are part of UCSF which has over 27,000 employees, the clinical settings differ in several ways (Figure 2).

#### Care at Home

CAH provides longitudinal primary and palliative care to homebound older adults in San Francisco, California. Patients enrolled in this practice are 65 years old or older and are unable to leave their homes due to dementia or other physical disabilities. The CAH team consists of physicians, nurse practitioners, social workers, nurses, and care coordinators who visit patients at home to offer support and treatment. Clinicians on the CAH team are specialists in geriatrics and/or palliative medicine.

#### Memory and Aging Center

MAC is a tertiary academic interdisciplinary clinic (involving neuropsychology, nursing, social work, and behavioral neurology) serving adults with suspected or diagnosed neurodegenerative disorders. There are over 40 clinicians who provide variable clinical time, with a large research component as a focal point of the clinic. Within MAC, there is a sub-clinic that aims to improve dementia-related symptom management, and it is within this sub-clinic that this pilot was undertaken. Referrals are received from all over the United States, with a focus on California. Patients referred are usually seen on a short-term basis to establish a diagnosis; however, a smaller percentage of patients are followed longitudinally once diagnosis is confirmed.

### Enrollment/Recruitment

This study received approval from the Institutional Review Board as projects 17-23299 and 20-31269. The commercially available GrandPad tablets underwent and passed an evaluation with UCSF’s IT Security Risk Assessment team.

#### Care at Home

**Figure 3.**
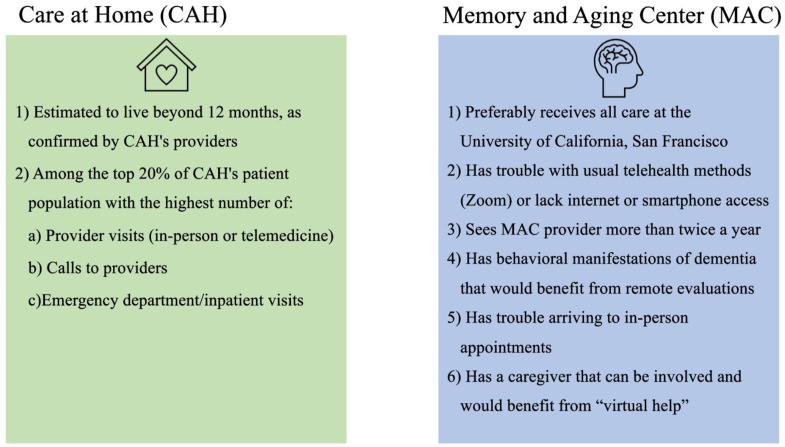
Selection criteria by site.

With the help of the clinicians, the research team identified participants from the top 20% of the CAH patient population who most utilized the healthcare system. Researchers defined “high use” as the highest number of in-person (home)/telemedicine provider visits, calls to providers, and ED/inpatient visits (Figure 3). A random sample of those “high use” patients were called by the CAH healthcare navigator to assess interest in participating in the study. The first 20 people that verbally agreed to participate were consented, given a telehealth readiness survey (Appendix Table 1) to understand their baseline access to telehealth and their previous experience with telephone visits, and subsequently trained to use the GrandPad in their home. Enrollment took place from May 2018 to February 2019 (Figure 1).

The UCSF Office of Population Health funded the CAH pilot and provided GrandPads free of charge to 20 patients, ages 60 to 94 over a one-year period. The focus was on conducting a feasibility study within the scope and funding constraints, rather than achieving a specific sample size based on statistical power calculations. Research participants were community-residing, predominantly low-income, and homebound (Table 1).

#### Memory and Aging Center

GrandPads were provided free of charge to a convenience sample of patients and caregiver dyads (*N* = 28) as a way to overcome the digital divide or potential access issues and to address neurobehavioral symptoms between visits. Researchers intentionally collected a convenience sample to mimic real-world implementation scenarios. Participants were selected by two clinician-investigators at MAC to meet selection criteria (Figure 3). To increase recruitment, the research team hosted two presentations and sent an email to MAC providers to explain the study, and enrollment criteria were loosened to include patients who did not have their primary care primarily at UCSF and who did not have major neurobehavioral symptoms.

At MAC, both patients and their caregivers provided consent. In cases where patients’ cognitive impairments prevented them from giving ethical consent, caregivers consented on their behalf. Enrollment took place on a rolling basis from December 2020 to March 2021 (Figure 1). Patient age ranged from 51 to 97 with an average of 74 years old (Table 1), all with cognitive impairment and MoCA scores ranging from 5 to 25 (Yokomizo et al., 2014). Participants were given a telehealth readiness survey (Appendix Table 1) to understand their baseline access to telehealth and their previous experience with telephone visits. They resided in California across a geographic range spanning 280 aerial miles north of San Francisco and 120 aerial miles south of San Francisco. Accordingly, devices were shipped directly to participants from GrandPad. Of the twenty-eight dyads (patient and caregiver pairs), eight disenrolled and one patient passed away after baseline measurements.

**Table 1.** Demographics by Site.

Characteristics		Care at Home (CAH)	Memory and Aging Center (MAC)
		(*N* = 20)	(*N* = 28)
Age (Avg ± S.D)		80.9 ± 11.0	74.3 ± 11.2
Gender	Female	10 (50%)	16 (57.1%)
	Male	10 (50%)	12 (42.9%)
Lives alone?	Yes	7 (35%)	16 (57.1%)
	No	13 (65%)	11 (39.3%)
	No response	0	1 (3.6%)
Ethnicity	Hispanic or Latino	1 (5%)	7 (25.9%)
	Not Hispanic or Latino	19 (95%)	20 (74.1%)
Montreal Cognitive Assessment (MoCA) Range		[4, 30]	[5, 25]
MoCA (Avg ± S.D)		18.8 ± 6.9	17.9 ± 5.3
Has tried telephone visits before with provider?	Yes—worked well	2 (10%)	20 (71.4%)
	Yes—but didn’t work	0	4 (14.3%)
	No—have not tried	18 (90%)	4 (14.3%)

*Note*: The average and standard deviation of caregiver age for MAC are 58.9 and 13.2, respectively.

### Measurement and Analysis

Our research team grounded implementation analysis in the Consolidated Framework for Implementation Research (CFIR)—a common implementation science framework looking at elements for successful implementation of interventions (Damschroder et al., 2022). In our study, GrandPads were first implemented in the CAH in 2018, with surveys designed to understand participant characteristics, utilization, and satisfaction. These surveys were then adapted for use in MAC in 2020 to gather similar insights, which we analyzed through CFIR for both clinical settings.

CFIR consists of five major domains: innovation (the “thing” being implemented—e.g., new treatment or device), outer setting (the setting in which the inner setting exists—e.g., a hospital system), inner setting (setting in which the innovation is implemented—e.g., team and clinic within a hospital system), characteristics of individuals involved (the roles of individuals—e.g. leaders and implementation facilitators), and the implementation process (activities and strategies used to implement the innovation). To inform implementation goals, we relied on the knowledge and experience of healthcare experts including the primary care site’s medical director (who is a clinician that also served as an implementation leader) and clinician-investigators working under the dementia specialty clinic. The authors used the CFIR domains as a framework for assessing the factors that facilitate implementation and present barriers to the GrandPad tablet across the two clinical sites. All patient-reported data was collected and stored in REDCap.

#### Care at Home

Surveys were distributed once to providers electronically at CAH to assess provider use of GrandPad for telehealth visits and perceptions on use for their patients. This pilot feasibility study was not designed to control for the number of patients using the GrandPad under each provider’s care. Our primary aim was to identify barriers and facilitators to the implementation of the GrandPad, rather than to determine the ultimate effectiveness of the telemedicine intervention. We acknowledge that differences in provider exposure to the GrandPad could exist, but this study focused on gathering insights into the implementation process.

Patients were asked to complete surveys by phone at baseline, three, six, nine, and twelve months. Surveys included demographic measures, a telehealth readiness survey, and satisfaction questions (Table 2 and Appendix Table 1). MoCA scores were collected from the electronic health record system for participants of CAH. For this study, we focus less on the individual patient outcomes and focus instead on the implementation process.

**Table 2.** Satisfaction with GrandPad by Site.

Questions	Care at Home (CAH) (*N* = 20)	Memory and Aging Center (MAC) (*N* = 28)
The GrandPad was easy to use.	Yes (85%)	Yes (68%)
The GrandPad helps me connect with my providers’ office.	Yes (80%)	No (63%)
It was easier for me to see my provider because of telemedicine.	Yes (70%)	No (68%)
I would prefer to see the provider “in person.”	Yes (80%)	Yes (42%)
I was uncomfortable using the GrandPad technology.	No (75%)	No (63%)

*Notes*: Yes includes Agree or Strongly Agree responses on a 5-point Likert scale.

No includes Disagree, Strongly Disagree, and Does Not Apply.

Participants’ last recorded responses were used:

CAH: 15 at 12 months; 2 at 9 months; 2 at 3 months.

MAC: 16 at 6 months; 3 at 3 months; 9 disenrolled or were lost to follow-up.

#### Memory and Aging Center

Patient and caregiver dyads were surveyed by phone at baseline, three and six months, and at disenrollment when possible. The initial study was planned as a 12-month study. However, due to changes in funding, the study was shortened to six months, and the number of participants was decreased prior to the start of enrollment. Patients and caregivers were each individually asked questions. For patients whose cognitive impairment led to caregivers providing consent on their behalf during enrollment, only the caregivers answered the survey questions. MAC providers and staff were given provider utilization and perception surveys similar to those for CAH.

Surveys utilized a mixture of structured and validated measures and included the opportunity for qualitative responses using open-ended questions. Validated measures included demographics, telehealth readiness, usage, and satisfaction with the device (Table 2 and Appendix Table 1). MoCA scores were collected from the electronic health record system for MAC participants. For this implementation analysis, we again focus less on the individual patient outcomes and focus instead on the implementation process.

## Results

The average age of CAH participants was 80.9 years old versus 74.3 years old in MAC participants, as shown in Table 1. The average and standard deviation for the age of MAC caregivers are 58.9 and 13.2, respectively. At CAH, 50% of participants were female and 50% male. At MAC, 57% of participants were female and 43% male. More than half (57%) of MAC participants lived alone, and 35% of CAH participants lived alone. CAH participants saw 5% of its participants self-identifying as Hispanic or Latino while 26% of MAC participants self-identified as Hispanic or Latino. MoCA scores ranged from a minimum of 5 to 25 for MAC and 4 to 30 for CAH, with means of 17.9 (standard deviation 5.3) and 18.8 (standard deviation 6.9), respectively. For CAH, which delivered the GrandPad before the pandemic, 90% of the participants had not tried telephone visits before with a provider at baseline, while 14% for MAC participants had not tried telephone visits before with a provider at baseline.

**Figure 4.**
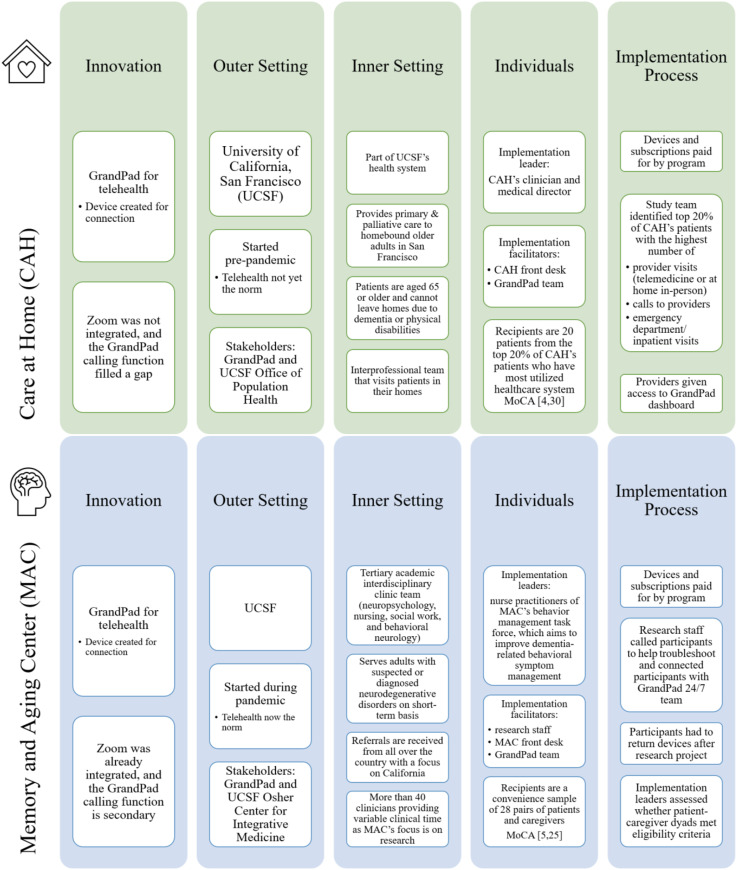
CFIR framework-informed implementation analysis by site.

The use of the GrandPad for clinical use in MAC was very limited, yet some participants still used the GrandPad for social or personal uses, with 18% of MAC participants using it for games, music, and other media. While both implementations occurred within UCSF, there were differences in study timing as implementation in CAH began before the pandemic at a time when telehealth was not yet the norm, and the implementation at MAC began during the pandemic when telehealth had been more established. The factors affecting overall implementation success are displayed in Figure 4 and described in detail within each CFIR domain and by case site.

### Care at Home

#### Innovation

While the GrandPad was initially created to encourage social connection within families, this study implemented the GrandPads for telehealth *before* telehealth was widely used at UCSF. During initial implementation in 2018, the GrandPad video call function filled a gap in the pre-pandemic era where telehealth was not yet standard. Additionally, some patients did not have access to tablets or computers, did not have data plans, or did not have the cognitive skills to install and use Zoom which was becoming the preferred platform. The determination of patients’ skills was based on baseline telehealth readiness surveys (which included questions about their previous experience with technology and their ability to use it independently) as well as healthcare providers’ assessments and interactions with patients. The GrandPad allowed providers to connect remotely with some of the homebound patients and could help with practice efficiencies.

#### Outer Setting

The UCSF Office of Population Health funded the pilot, with the hypothesis that providing age-friendly tablets would increase CAH’s ability to conduct telehealth visits within hard-to-reach populations. Funders also hoped to see a decrease in healthcare utilization for ambulatory sensitive conditions. This could also potentially increase the institution’s revenue for telehealth visits as opposed to lower revenue from incomplete visits or telephone-only interactions. This financial support and innovative idea were critical to implementation.

#### Inner Setting

CAH included providers who were at the forefront of starting telehealth before it was mainstay due to a smaller palliative care telehealth pilot in the prior years. The patients served in CAH are traditionally left out of telehealth because of the digital divide and/or cognitive impairment that presents barriers to use. The interest in telehealth before implementation of the GrandPad and the nature of home-based care serving hard-to-reach populations were key success features.

#### Individuals

All members of the clinical team were involved in implementation in some capacity, including the medical director who served as the implementation lead. The front desk staff and care navigator were instrumental in enrolling participants, delivering the GrandPad and helping with initial training. Healthcare providers ultimately initiated visits with patients via a GrandPad portal. The providers were involved in the use of the GrandPad portal to connect with patients for remote visits. The identification of the recipients, the support of the front desk, and participation of the clinicians came together within the site and created a coordinated implementation effort.

#### Implementation Process

Devices and subscriptions for the innovation were paid for by CAH via the UCSF Office of Population Health. The care navigators and front desk team were critical in helping patients receive their GrandPad and troubleshooting with the GrandPad team if there were set-up issues. The medical director and implementation lead helped providers with access to the GrandPad portal and served as a liaison with the GrandPad team if there were access issues. Ultimately, the CAH implementation process was an internal effort that included the medical director, the front desk, and the GrandPad team from the beginning of the implementation.

### Memory and Aging Center

#### Innovation

The GrandPad device was introduced to MAC in 2020. Prior to the pandemic, telehealth was not a routine part of this clinic. When this study was approved and designed, telehealth was not standard, such that GrandPad integration was thought to be very innovative. The COVID-19 pandemic spurred the integration of Zoom into health systems, and telehealth quickly became standardized to enable safe and remote treatment. As such, the GrandPad video call function became secondary to the system-integrated Zoom, and participants and their caregivers preferred to use already familiar technology over the GrandPad video call function when they connected with their provider. Of note, patients at MAC tended to not connect without their caregiver. Without a functioning telehealth option, there was no “visual” way to assess patients exhibiting neurobehavioral visits symptoms between visits.

#### Outer Setting

The outer setting is the same as the first clinical setting (UCSF), and the implementation at MAC received funds from the UCSF Osher Center for Integrative Medicine. However, the MAC implementation did not have the same level of financial support that the CAH implementation received from the UCSF Office of Population Health. Additionally, the MAC implementation faced the most critical event disrupting implementation: the COVID-19 pandemic, which forced the outer setting to integrate Zoom and consequently narrow the telehealth gap that the GrandPad was intended to address at the time of study approval. Thus, it is unclear if some of the decreased clinical follow-up was due to the pandemic directly or other factors.

The social distancing aspect of the pandemic also resulted in a change in process as devices were mailed directly to the participants at their homes by GrandPad directly. At the time of implementation, Zoom had become the standard telehealth option across UCSF such that all processes for telehealth were related to Zoom, integrated into the electronic medical record, and the use of other platforms required institutional approval. As such, much of the institutional attitudes and support of telehealth were focused on one platform to reach the largest number of patients, without a focus on what to do about those who do not have access to Zoom or have no other way to use telehealth.

#### Inner Setting

MAC serves adults with suspected or diagnosed neurodegenerative disorders on a short-term or intermittent basis, with fewer patients having longitudinal care. As a result, there was less continuity for patients and the provider pool for the study was larger given a larger number of clinicians. There was a strong desire by the neurobehavioral sub-clinic to be able to reach their patients and visually evaluate behavioral symptoms. This strong desire contributed to the positive attitudes toward this implementation. However, as stated above, because the GrandPad needed a separate workflow which required the providers to have different logins and not just use of Zoom, this may have made it difficult to better integrate the GrandPad into scheduling and clinical workflows.

#### Individuals

Two nurse practitioners who lead a neurobehavioral symptom sub-clinic within MAC (specifically for patients with dementia-related behavioral symptoms) functioned as implementation leaders for this study by helping to integrate the GrandPad into practice. MAC’s front desk staff, the UCSF research team, and the GrandPad team served as implementation facilitators and were the key individuals involved. MAC’s front desk staff assisted in providing GrandPad portal instructions for providers and patients in the electronic healthcare system. The research staff members called participants to help troubleshoot the device and connect participants with the GrandPad tech team. The nurse practitioners were key to identifying appropriate participants and communicating with providers about the project. However, their roles did not allow them to significantly change clinical workflows regarding the use of telehealth. There was no clinical leadership involved in implementation, which may have also contributed to challenges with implementation.

#### Implementation Process

Devices and subscriptions were paid for by the UCSF Osher Center for Integrative Medicine, and participants were asked to return devices after completion of the project. The research staff called participants, helped troubleshoot, and connected participants with a GrandPad team. MAC’s implementation team coordinated with the implementation team at the other site. Having dedicated staff to help with troubleshooting and the correct identification of dyads was critical. As noted above, the nurse practitioners were also key in identifying the needs of patients and those who could most benefit from the GrandPad and in engaging the providers.

### Satisfaction

To evaluate overall satisfaction of the participant sample, we focused on participants’ last recorded responses to 5 satisfaction statements (Table 2). While the participants ranked the statements on a 5-point Likert scale, we considered “Agree” or “Strongly agree” responses as “Yes” while considering “Disagree,” “Strongly disagree,” and “Does not apply” as “No.” For the statements “The GrandPad helps me connect with my providers’ office.” and “It was easier for me to see my provider because of telemedicine.” the majority of CAH participants answered “Yes” while the majority of MAC participants answered “No.” All other statements saw the same overall answers for the majority or plurality of participants at each site.

#### Care at Home

Each of the 20 CAH participants was surveyed every three months for one year. Overall, the participants said “Yes” to the following statements: the GrandPad was easy to use (85% of the site’s participants); the GrandPad helps me connect with my providers’ office (80%); it was easier for me to see my provider because of telemedicine (70%); and I would prefer to see the provider “in person” (80%). Of the site’s participants, 75% said “No” to the statement “I was uncomfortable using the GrandPad technology.”

#### Memory and Aging Center

Each of the 28 MAC participants was surveyed every three months for six months. Sixteen of the responses were from the six-month point, three from the three-month point, and nine disenrolled or were lost to follow-up. Overall, the participants said “Yes” to the statements “the GrandPad was easy to use” (68% of the site’s participants) and “I would prefer to see the provider ‘in person’” (42%). The participants said “No” to the statements “The GrandPad helps me connect with my providers office” (63%); “It was easier for me to see my provider because of telemedicine” (68%); and “I was uncomfortable using the GrandPad technology” (63%).

Eight dyads disenrolled throughout the study (mostly around the three-month mark) due to difficulties in adjusting to the GrandPad, including challenges with downloading items despite the availability of the family administrator option. Additionally, the caregivers of the four dyads who disenrolled shared that they found the GrandPad less intuitive to use compared to their primary devices (such as laptops, iPads, or iPhones) leading many to return to their familiar technology. For one caregiver, the cultural and contextual factors also played a role, such as the lack of games tailored to the Latino population and concerns around privacy, further contributing to the decision to discontinue use of the GrandPad with the participant.

## Discussion

Healthcare’s technological landscape continuously evolves, making telehealth accessibility critical to prevent a wider digital divide and associated healthcare cost burdens and health inequities. Older adults, homebound individuals, and those with cognitive impairments often face challenges adapting to telehealth due to limited familiarity or lack of necessary tools like devices and broadband access. Through two pilot programs, our study focused on these patient populations because they stand to benefit significantly from telehealth designed for older adults. Both pilots occurred at critical points, one immediately pre-pandemic when telehealth was not standard nor was it universally reimbursed, and the other during the pandemic, when telehealth became the mainstay of clinical care and when older adults were disproportionally affected by COVID-19 in terms of healthcare access, morbidity, and mortality. We aimed to understand if technology designed for older adults could facilitate uptake and implementation of telehealth for those with limited telehealth access or cognitive impairment.

Our overall results demonstrate differences in use and acceptability across the two sites, with increased success with clinical integration of GrandPad in the CAH population. Specifically, the care setting (inner setting), willingness and motivation to use new technology (inner setting and individuals), and, most critically, the implementation process are all at the center of successful implementation. Participants’ responses to “Have you tried telephone visits before with your provider?” differed significantly by site, with 90% of CAH participants responding “No” compared to 14% of MAC participants. This difference in telehealth readiness influences the differences in participants’ willingness and motivation to use new technology, with CAH showing potentially higher willingness and motivation to adopt the GrandPad to address the telehealth care gap in their lives. Additionally, regarding outer setting, the parent organization’s willingness to fund the CAH pilot was crucial for providing GrandPads free of charge to users and ensuring cost was not a barrier to participation. CAH’s decision to continue funding the tablets after the conclusion of the pilot contributed to sustainability of the implementation.

Results also demonstrate that the study’s timing influenced implementation success. The GrandPad was intended to provide a more reliable telehealth solution to cognitively impaired individuals. However, the pandemic makes it difficult to see if this device could be successfully used in a dementia clinic more effectively. At their baseline during the pandemic, MAC participants demonstrated more telehealth experience than participants from CAH. At their baseline before the pandemic, CAH participants lacked the same level of telehealth experience, which may have contributed to their willingness to learn a new device.

In CAH’s implementation, which began prior to the pandemic, telehealth was not uniformly available. The GrandPad video call function filled a needed gap, and Zoom was not available on it at the time. Both providers and patients were motivated to connect between visits when no standard approach existed. GrandPad also allowed patients to connect with providers independently without a family member or caregiver. At MAC, the GrandPad was implemented in 2020, when telehealth was already the norm, and UCSF’s healthcare system had integrated Zoom into standard workflows due to the COVID-19 pandemic. Therefore, the GrandPad’s video call function was not filling a significant gap, possibly leading to decreased motivation for its use among patients and providers.

Caregiver presence may have influenced implementation at MAC. Initially, we hypothesized that caregiver involvement would enhance GrandPad usage by MAC patients and caregivers for telehealth and non-telehealth purposes. While caregivers initially supported using GrandPads, many caregivers eventually found their primary devices (e.g., iPads or iPhones) to be more intuitive and culturally familiar. This shift likely influenced the overall engagement and effectiveness of the tablet intervention for MAC.

The differences in sites also played a crucial role in implementation efforts (inner setting). While CAH provides primary and palliative care to homebound older adults, MAC provides less continuous, consultative services to adults with suspected or diagnosed neurodegenerative disorders. A smaller number of providers at CAH coupled with the longitudinal aspect of care may have resulted in both providers and patients being more invested in the intervention. Greater investment and longer-term care facilitated implementation and more telehealth use of the GrandPad. Lastly, GrandPad was intended to provide a more reliable way to provide telehealth to cognitively impaired individuals, but low usage and pandemic effects make it difficult to assess its effectiveness in a dementia clinic under different circumstances. We did not examine changes in healthcare utilization (despite this being a hypothesis) or differences in symptom burden due to the small sample size and vast implementation differences between the sites.

In this study, random and convenience sampling differences may have impacted the results. We used random sampling to select study participants among CAH’s high-use patients, ensuring a more representative sample and enhancing the generalizability of the findings. In contrast, we used convenience sampling to recruit participants at MAC, which may introduce selection bias and skew results towards participants more open to technology. These recruitment method differences should be considered when interpreting the comparative effectiveness and user satisfaction of the GrandPad across the two settings.

In summary, barriers to implementing an age-friendly device like the GrandPad include several factors. First, the presence of an existing system-integrated telehealth platform needs consideration. If such a platform already exists, it must be explained that different workflows and platforms may be needed for those without broadband access, devices, or the ability to log on to the standard platform to ensure they are not left behind. Secondly, the success of GrandPad implementation may depend on continuity of care. Clinics with long-term and regular patient interactions are more likely to succeed in implementing the GrandPad. Considering these barriers when implementing accessible telehealth devices like the GrandPad will help prevent significant portions of patient populations from being left behind as technology advances.

## Conclusion

This study is the first of its kind to use a tablet *specifically* designed for older adults and determine if it is feasible to implement as a telemedicine tool in clinics serving predominantly older adults and those with cognitive disorders. Our findings indicate that while most participants found the GrandPad easy to use and were comfortable with the technology, there were mixed results on the ability to connect with providers and the preference for in-person visits over telemedicine. We conclude that populations that might benefit from telemedicine with the GrandPad include users struggling with cognitive impairment, decreased visual or manual dexterity, or a lack of a caregiver to help with complex telehealth platforms. The device and its accompanying data plan may also be useful for those lacking broadband access.

Telehealth has become a standard component of care in many healthcare settings; however, some patient populations lack the means and understanding to own or operate specific telehealth requirements like devices and broadband. Addressing this issue requires recognizing that the onus of implementing health care is not solely the responsibility of patients but also healthcare systems. By acknowledging disparities in telehealth access and taking proactive measures to bridge the gap, we can fulfill our duty to care for the entire population, not just those who can easily engage and keep up with ever-evolving telehealth tools.

## Supplemental Material

Supplemental Material - The Use of Age-Friendly Technology in the Care of Older Adults: Two Implementation Case Studies Supplemental Material for The Use of Age-Friendly Technology in the Care of Older Adults: Two Implementation Case Studies by Soe Han Tha, Katrina Hough, Nhat Bui, Sarah Dulaney, and Carla Perissinotto in Journal of Applied Gerontology.
