# Supplementary material for: The Use of Age-Friendly Technology in the Care of Older Adults: Two Implementation Case Studies
Source: J Appl Gerontol. 2025 Jun 24;45(3):515–26. doi: 10.1177/07334648251343513 (PMC12876409; doi:10.1177/07334648251343513)
Supplement: Supplemental Material - The Use of Age-Friendly Technology in the Care of Older Adults: Two Implementation Case Studies [file sj-pdf-1-jag-10.1177_07334648251343513.pdf]

APPENDIX

Table 1: Telehealth Readiness Questions

Assessment Area	Survey, questions, and response options
Telehealth Readiness	Telehealth Readiness Survey<br>• Have you tried telephone visits before with your provider?<br>• Do you or a caregiver have a computer, tablet, or smartphone?<br>• What kind of technology do you have in your home?<br>• Does your desktop have a camera?<br>• Does your desktop have speakers?<br>• Does your desktop have a microphone, or do you have a headset that includes a microphone?<br>• Does your laptop have a camera?<br>• Does your laptop have speakers?<br>• Does your laptop have a microphone, or do you have a headset that includes a microphone?<br>• Do you have a high-speed (or broadband") Internet connection in your home?<br>• Do you have wireless (Wi-Fi) Internet access in your home?<br>• Do you have a quiet, private space at home where you could sit to participate in a video appointment with your physician?<br>• For caregivers only: Would you or another caregiver want or need to be present during the visit in order to work the video conference?
